# Widespread pain syndrome in long COVID-19: melatonin as an adjuvant treatment

**DOI:** 10.3389/fpain.2025.1609095

**Published:** 2025-06-04

**Authors:** Amine Souissi, Pablo Prieto-González, Helmi Ben Saad

**Affiliations:** ^1^ Faculté de Médecine de Sousse, Hôpital Farhat HACHED, Laboratoire de Recherche (Insuffisance Cardiaque, LR12SP09), Université de Sousse, Sousse, Tunisie; ^2^ Sport Sciences and Diagnostics Research Group, GSD-HPE Department, Prince Sultan University, Riyadh, Saudi Arabia

**Keywords:** antioxidants, chronic pain, inflammaging, long COVID-19, melatonin, oxidative stress, widespread pain syndrome

## Abstract

Long coronavirus disease 2019 (LC19) represents a complex global health challenge. Survivors frequently report persistent problems like widespread pain syndrome (WPS), cognitive dysfunction, cardiovascular complications, and sleep disturbances. These health problems, which are worsened by oxidative stress and inflammaging, open the prospect of treatment strategies targeting these mechanisms. Melatonin is a potential option for treating LC19 problems because of its anti-inflammatory, antioxidant, and pain-modulating properties. Melatonin targets shared pathological pathways, offering a promising approach to reducing inflammation, oxidative stress, and neuroendocrine dysfunction. The present mini-review explores the therapeutic potential of melatonin in the treatment of LC19, focusing on its effects on WPS and inflammation.

## 1 Introduction

Long coronavirus disease 2019 (LC19) has been recognized by the World Health Organization as a global health crisis, warranting “emergency use” attention (1). Affecting over 60% of COVID-19 survivors (2), LC19 is characterized by a variety of chronic symptoms, including exhaustion, anxiety, depression, sleep disturbances, cognitive impairments, widespread pain, and systemic inflammation (3–7). Despite extensive research into acute COVID-19 complications, the long-term sequelae of LC19 remain poorly understood, highlighting the urgent need for studies to unravel its mechanisms and identify effective therapeutic strategies (4–7). Among the diverse symptoms of LC19, widespread pain syndrome (WPS) (8) and systemic complications such as myocarditis (9), are the most alarming. WPS is marked by chronic, diffuse musculoskeletal pain, disturbing patients’ comfort and satisfaction (8). Both conditions share a key pathological process: inflammaging, a chronic inflammatory state traditionally associated with aging (10), but increasingly linked to post-infectious syndromes (8, 9).

Emerging evidence suggests a critical role of altered endogenous melatonin levels in driving oxidative stress and systemic inflammation in LC19 (9). The ability of melatonin to counteract inflammation positions it as a potential therapeutic strategy for addressing the systemic effects of LC19, including WPS. This review explored the therapeutic potential of melatonin in addressing LC19's multifaceted challenges, particularly WPS and the systemic inflammation associated with the condition. We suggest that therapies focusing on these interconnected pathways, such as melatonin supplementation, could be promising for minimizing the complex consequences of LC19.

## 2 Inflammaging and its role in long COVID-19 complications

One of the main causes of health problems in LC19 is inflammation (9), which is exacerbated in elderly individuals due to ‘inflammaging’. The latter is a condition where aging leads to a chronic, low-grade inflammatory state (10). This condition is characterized by a sustained pro-inflammatory environment fueled by persistent oxidative stress, immune dysregulation, and the overproduction of pro-inflammatory cytokines (9, 10). In LC19, these interconnected mechanisms play the major role in the progression and severity of symptoms, including WPS (8) and cardiovascular complications (e.g., myocarditis) (9). In LC19 patients, inflammation is believed to amplify musculoskeletal pain through both central and peripheral sensitization of pain pathways (11). Chronic inflammation can disrupt the normal functioning of sensory neurons, increasing their excitability and amplifying pain signals (12). Furthermore, the persistent inflammatory milieu can interfere with the neuroendocrine system, impairing the production and regulation of melatonin (13). Consequently, chronic inflammation can induce sleep problems, potentiate oxidative stress and systemic inflammation, and disrupt the healing process (9). Interestingly, sleep disturbances can, in turn, activate inflammatory mechanisms and exacerbate pain (7, 14, 15).

Aging-related declines in endogenous defenses further promote the negative effects of inflammation in LC19 patients (particularly in elderly people suffering from inflammaging) (9, 10). For example, decreased melatonin production (i.e., a sign of aging) is associated with decreased antioxidant activity and an inability to counteract the oxidative stress and pro-inflammatory signaling associated with WPS (16, 17). Importantly, this decline in melatonin levels induces health complications and contributes to systemic symptoms, including fatigue, sleep disturbances, and cognitive impairments (9).

## 3 Widespread pain syndrome in long COVID-19

It is well established that WPS is considered a severe complication affecting LC19 survivors, with a prevalence ranging from 10% to 80% (8). WPS is characterized by diffuse and chronic musculoskeletal pain, most likely results from a combination of immune system dysregulation, neuroinflammation, and central sensitization (12). Importantly, oxidative stress, decreased melatonin levels, and irregular circadian rhythms can bring on these health difficulties (18, 19). In LC19, persistent inflammation and the hyperactivation of pain signaling pathways may perpetuate WPS, leading to long-term physical and psychological impairments (12).

Analgesics are commonly used to manage pain in WPS (20), however, they primarily provide symptomatic relief without addressing the underlying causes of the condition and are often associated with adverse side effects (20). Conversely, melatonin, which is naturally produced by the body, holds promise as a safer alternative due to its ability to reduce oxidative stress, regulate circadian rhythms, and modulate systemic inflammation (9). Despite its frequency, WPS is still poorly understood in LC19 research, emphasizing the necessity for a multifaceted therapeutic strategy that treats localized and systemic symptoms. There are still few evidence-based treatments approaches for WPS in this population, which underscores the need for additional focused studies in this field.

The interesting 2025 letter to the editor entitled “potential of melatonin as a treatment option for long COVID: A call for research” highlights the *urgent* need to address the persistent and multifaceted challenges posed by LC19, emphasizing the potential role of melatonin as a therapeutic agent (21). Kakad et al. (21) skillfully describe the mechanisms of action of melatonin, such as its capacity to regulate neuroinflammation, oxidative stress, and mitochondrial dysfunction. The aforementioned three important pathogenic processes underlie the systemic and neuropsychiatric symptoms of LC19 (21). Interestingly, the letter highlights the overwhelming data supporting melatonin's advantages, as proven in past studies on acute COVID-19 (22), and advocates for dedicated clinical research to examine its efficacy in LC19 patients (9). Inspired by this essential request, we were compelled to create the present mini-review to reaffirm the importance of melatonin in controlling LC19. Our review supported the letter's observations (21), and emphasizes the urgency of taking quick action. In order to reduce the burden of LC19, we support putting this increasing body of evidence into clinical practice and implore the scientific community to give melatonin's therapeutic potential top priority. This will open the door to more focused and easily accessible therapies.

## 4 Melatonin as a therapeutic target in long COVID-19

Melatonin, an endogenous neurohormone with strong antioxidant, anti-inflammatory, and immunomodulatory properties (23), shows great potential as an adjuvant treatment for LC19 (9). Melatonin, which is well-known for controlling circadian cycles and mitochondrial activity, can counteract many mechanisms underlying LC19 complications, including weight gain (24), widespread pain (16, 17), sleep disturbances (7, 9), and myocarditis (9).

Therefore, research on melatonin's function in LC19 management is an important and pertinent public health project. It is critical to recognize that exogenous melatonin may harm some groups, including nursing mothers (25). Additionally, because of the uncertain long-term endocrine consequences, prolonged or high-dose melatonin usage should be avoided (26). Thus, expert advice and evidence-based recommendations are crucial to optimize the use of melatonin and improve endogenous production (9).

### 4.1 Inflammaging, pain modulation, and widespread pain syndrome

**Figure 1:**
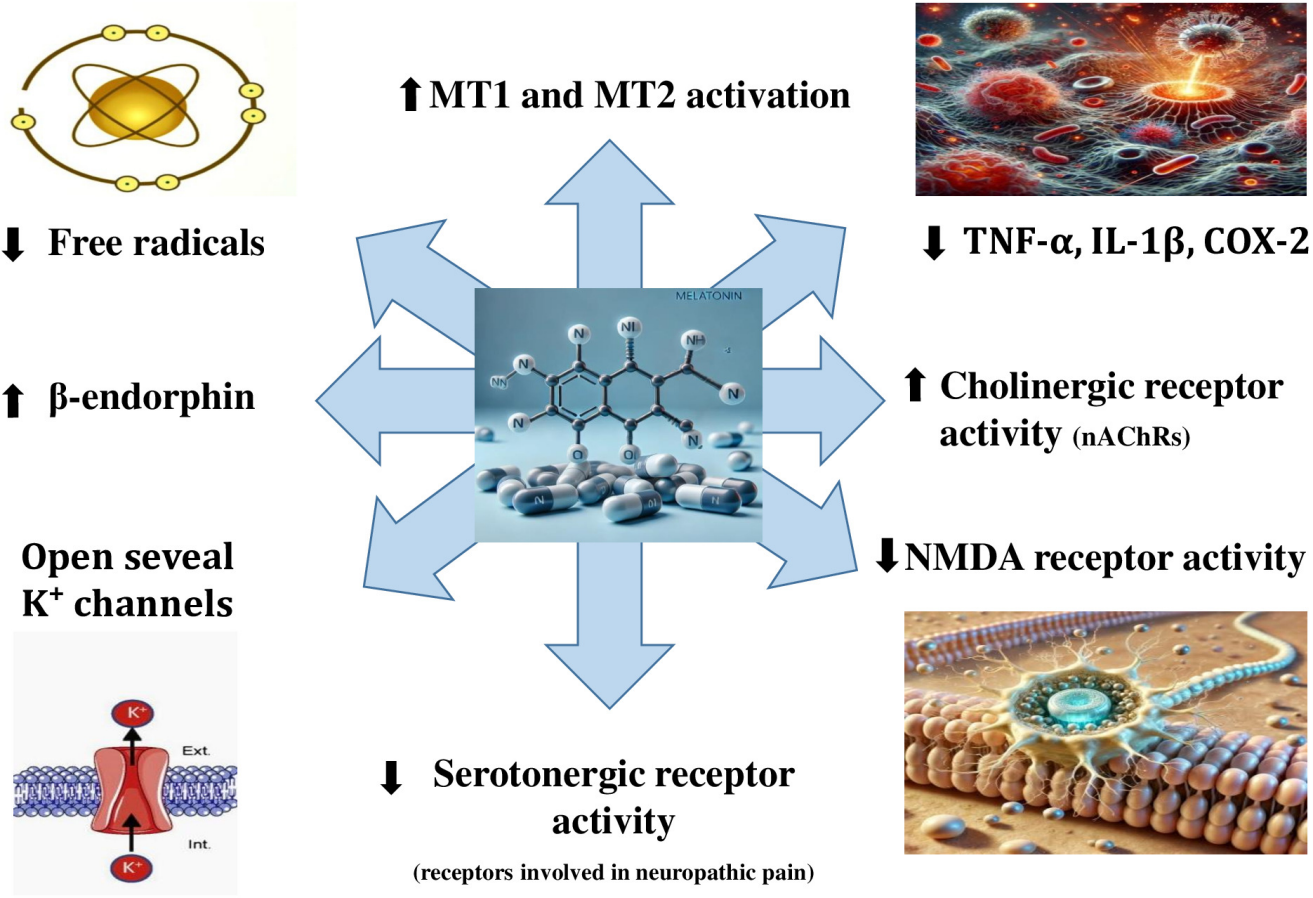
Mechanisms underlying melatonin's analgesic effect. **COX-2**, cyclooxygenase-2; **IL-1β**, Interleukin 1 β; **K^+^**, potassium ion; **MT1**, melatonin receptor 1; **MT2**, melatonin receptor 2; **NMDA**, N-methyl-D-aspartate; nAChRs, nicotinic ACh receptors; **TNF-α**, tumor necrosis factor α.

Melatonin emerges as an effective adjuvant treatment for LC19 due to its capacity to inhibit both the primary causes of chronic pain and inflammation (27). Melatonin is a powerful antioxidant and anti-inflammatory molecule that inhibits the synthesis of pro-inflammatory cytokines, including interleukins 6 and 1β, and tumor necrosis factor α (TNF-α) (28), essential for the pathways leading to inflammation and pain (29). Melatonin mitigates oxidative damage in nociceptive tissues and restores redox balance by scavenging reactive oxygen species and boosting endogenous antioxidant defenses (30, 31). Interestingly, it has been shown that melatonin directly tackles the inflammatory cascades that underlie chronic pain and tissue dysfunction associated with aging by regulating “nuclear factor-kappa B” activity (32). Beyond its anti-inflammatory properties, melatonin modulates central pain mechanisms by influencing key pathways, such as the “N-Methyl-D-Aspartate (NMDA)” receptor and the opioid and serotonin systems (to modify central pain mechanisms) (31, 33). Consequently, this helps alleviate hyperalgesia and reduce central sensitization (34). Additionally, melatonin improves circadian rhythm regulation and sleep quality, indirectly reducing pain perception by attenuating sleep disturbances (a common exacerbating factor in WPS) (28). Furthermore, melatonin suppresses the activation of microglia and astrocytes in the central nervous system (35), which are key contributors to chronic pain sensitization (36). Interestingly, melatonin also exerts its analgesic actions by binding to opioid receptors and increasing the release of β-endorphin (37). Furthermore, melatonin can exert analgesic actions by increasing the activity of cholinergic receptors, especially the neuronal nicotinic acetylcholine receptors sensitive to α-bungarotoxin (38). The complex analgesic action of melatonin is simplified in Figure 1.

**Figure 2:**
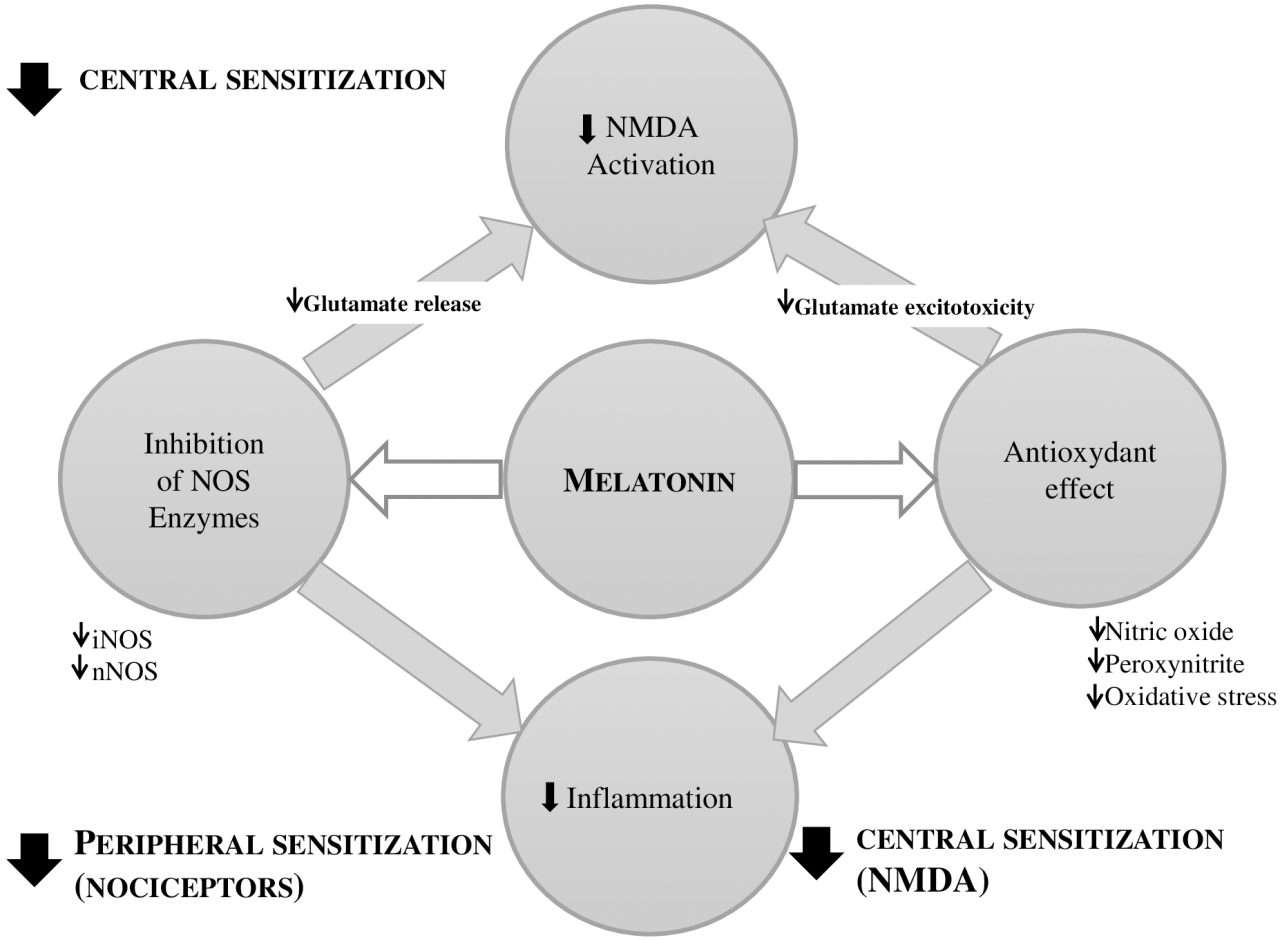
Role of melatonin in reducing central and peripheral sensitization via N-methyl-D-aspartate (NMDA) modulation and antioxidant effects. Melatonin reduces pain sensitivity and neuroinflammation by inhibiting inducible and neuronal nitric oxide (NO) synthases (iNOS, nNOS, respectively). It suppresses NO-mediated inflammation, scavenges peroxynitrite, and attenuates NMDA receptor overactivation. **iNOS**, inducible NOS ; **nNOS**, neuronal NOS ; **NOS**, nitric oxide synthase.

Pro-nociceptive effects, neurotoxicity, and neuroinflammation are all influenced by excessive nitric oxide (NO) production (32). Remarkably, melatonin reduces pain by inhibiting the activity of NO synthases (NOS) enzymes (i.e., inducible- and neuronal- NOS), which are linked to anxiety brought on by chronic pain. Melatonin suppresses NO-mediated inflammatory responses (32) and scavenges peroxynitrite, which has a potent oxidative stress effect and increases sensitivity (39). Furthermore, by reducing NO levels, melatonin indirectly suppresses NMDA receptor overactivation, which reduces pain sensitivity and hyperalgesia. Notably, melatonin is a strong anti-inflammatory and antinociceptive hormone that works especially well in settings with excess NO generation (Figure 2). To the best of the authors’ knowledge, we highlight for the first time that melatonin reduces pain-promoting effects via counteracting TNF-α-mediated inducible-NOS activation. We suggest that the antinociceptive effects of melatonin could be largely attributed to this mechanism.

Melatonin is a special and multifaceted treatment option for LC19 because it addresses the interconnected pathways of inflammaging and chronic pain (27). By targeting inflammation, oxidative stress, disrupted circadian rhythms, and central pain sensitization, melatonin holds the potential not only to manage widespread pain but also to improve overall systemic resilience in LC19 patients (9, 33). This multifaceted approach may significantly enhance recovery and quality of life in individuals suffering from WPS as part of the LC19 spectrum.

## 5 Conclusion

Targeting these pathways, melatonin is an attractive adjuvant therapy that offers several advantages in managing LC19. Melatonin can potentially counteract the chronic effects of widespread LC19 pain by restoring circadian rhythms, reducing inflammation, and minimizing oxidative damage. We propose that melatonin could be employed as a potential treatment for WPS induced by LC19. It is essential to carry out particular clinical research concentrating on the ideal dosage, safety, and effectiveness of exogenous melatonin in treating WPS induced by LC19 in order to firmly establish its place in clinical practice. These investigations ought to evaluate how melatonin affects LC19 patients’ pain management, cognitive impairment, and neuropsychiatric symptoms. In order to investigate the therapeutic potential of melatonin and open the door for evidence-based therapies in the treatment of protracted COVID, we therefore implore the scientific community to give priority to these clinical trials.
